# Knowledge and Compliance With Universal Precautions Among Nurses at a Tertiary Care Teaching Hospital in Central India

**DOI:** 10.7759/cureus.111995

**Published:** 2026-07-03

**Authors:** Vijay K Goad, Deepak Makode, Harshita G Pawar, Raj P Vaidya

**Affiliations:** 1 General Practice, Nandkumar Singh Chauhan Government Medical College, Khandwa, IND; 2 General Practice, All India Institute of Medical Sciences Bhopal, Bhopal, IND; 3 General Practice, Ram Krishna Medical College Hospital and Research Centre, Bhopal, IND; 4 Community Medicine, Government Medical College, Datia, IND

**Keywords:** compliance, healthcare-associated infections, infection control, nurses, personal protective equipment, universal precautions

## Abstract

Background

Universal precautions are essential infection control measures aimed at preventing healthcare-associated infections and occupational exposure among healthcare workers (HCWs). However, compliance remains suboptimal, particularly in resource-limited settings.

Objective

The aim of this study was to assess the knowledge and compliance regarding universal precautions among the nursing staff at a tertiary care teaching hospital and identify gaps in infection control practices.

Methods

A hospital-based cross-sectional study was conducted from March to April 2026 among 40 nurses at Government Medical College, Datia, Madhya Pradesh, India. Participants were selected using simple random sampling. Data were collected using a validated structured questionnaire assessing knowledge (15 items) and compliance (20 items on a Likert scale). Statistical analysis was performed using Jamovi, version 2.6.44 (The jamovi Project, Sydney, NSW, Australia).

Results

Among the 40 nurses included in the study, 31 (77.5%) were aged 24-30 years, and 24 (60.0%) had 1.5-5 years of work experience. Adequate knowledge regarding universal precautions was observed among 31 (77.5%) nurses, while five (12.5%) demonstrated very good knowledge scores. Knowledge regarding key infection control practices was high, with 40 (100%) participants aware of hand hygiene between patients and 39 (97.5%) reporting correct knowledge regarding glove use for blood collection and avoidance of needle recapping. However, 34 (85.0%) nurses correctly recognized that the primary objective of universal precautions is not limited solely to protecting HCWs, while five (12.5%) believed that such precautions were required only for infected patients. Compliance with hand hygiene practices was high, with 36-38 nurses (90.0%-95.0%) consistently adhering to recommended practices. Similarly, adherence to glove use during high-risk procedures was observed among 37-39 nurses (92.5%-97.5%). Lower compliance was observed for glove use during injections among 22 (55.0%) nurses and for apron and cap/shoe-cover use among 26-27 nurses (65.0%-67.5%). Additionally, 4 (10.0%) nurses reported non-adherence to safe needle recapping and post-exposure management practices.

Conclusion

Despite adequate knowledge, a notable knowledge-practice gap exists among nurses. Strengthening training, ensuring personal protective equipment (PPE) availability, and reinforcing monitoring mechanisms are essential to improve compliance and enhance infection control practices.

## Introduction

Infection prevention and control are fundamental components of healthcare delivery, ensuring patient safety while protecting healthcare workers (HCWs) from occupational exposure to infectious hazards encountered during clinical practice [1]. Universal precautions comprise a set of infection prevention guidelines designed to minimize the transmission of blood-borne pathogens, including human immunodeficiency virus (HIV), hepatitis B virus (HBV), and hepatitis C virus (HCV). Introduced by the Centers for Disease Control and Prevention (CDC) in 1985, these precautions recommend that all blood and selected body fluids be regarded as potentially infectious and handled using standard safety measures [2,3]. Owing to their frequent contact with patients, contaminated equipment, and biological specimens, HCWs remain at increased risk of acquiring occupational infections [4].

Healthcare-associated infections continue to pose a major challenge to patient safety worldwide. A recent global systematic review involving more than 29 million participants estimated an overall healthcare-associated infection prevalence of 14%, with the highest burden reported in the WHO African Region and intensive care units (ICUs) [5]. The prevalence of healthcare-associated infections varies from 5% to 40% across different regions and may exceed 30% of ICU admissions [6]. In low- and middle-income countries, the risk of acquiring healthcare-associated infections is estimated to be up to 20 times higher than in high-income settings. In India, pooled ICU surveillance has reported bloodstream infection rates of 5.3-7.3 per 1,000 patient-days, central line-associated bloodstream infection rates of 8.3-12.1 per 1,000 central line-days, urinary tract infection rates of 1.7-2.8 per 1,000 patient-days, and catheter-associated urinary tract infection rates of 8.3-12.1 per 1,000 catheter-days, highlighting the substantial burden of healthcare-associated infections and the need for strengthened surveillance and infection prevention strategies [7].

Universal precautions are central to preventing healthcare-associated infections and occupational exposure among HCWs. Their core components include hand hygiene, appropriate use of personal protective equipment (PPE), safe handling and disposal of sharps, and adherence to safe injection practices, all of which contribute substantially to reducing the transmission of infectious agents in healthcare settings [8].

Despite well-established guidelines, compliance with universal precautions remains suboptimal worldwide, particularly in resource-limited settings where inadequate training, limited availability of PPE, and heavy workloads compromise adherence. Studies have consistently reported deficiencies in hand hygiene, sharps disposal, and the appropriate use of PPE among healthcare professionals [9,10].

Although nurses generally demonstrate higher compliance than other categories of HCWs, important gaps in practice persist [11]. Adherence to universal precautions is influenced by multiple factors, including knowledge, institutional infection-control policies, workload, perceived occupational risk, and previous occupational exposure [10,12,13]. As tertiary care teaching hospitals function both as referral centers and training institutions, maintaining high standards of infection prevention is essential to reduce healthcare-associated infections, ensure patient safety, and protect the healthcare workforce [14,15].

In this context, the present study was undertaken at Government Medical College, Datia, Madhya Pradesh, India, to assess the knowledge and compliance with universal precautions among nursing staff and to identify gaps in infection control practices that could guide targeted interventions for improving infection prevention and patient safety.

## Materials and methods

Study design and setting

A hospital-based cross-sectional study was conducted from March to April 2026 among nursing staff at Government Medical College, Datia, Madhya Pradesh, India, as part of an institutional infection prevention and control audit. The study was reported in accordance with the Strengthening the Reporting of Observational Studies in Epidemiology (STROBE) guidelines to ensure methodological transparency and reproducibility. The primary objective was to assess knowledge and compliance with universal precautions among nursing staff, while the secondary objective was to identify specific areas of noncompliance requiring targeted infection control interventions.

Study population

The study population comprised registered nurses working at Government Medical College, Datia, who were directly involved in patient care, biomedical waste management, or infection prevention activities. Both male and female nurses fulfilling the eligibility criteria were considered for participation. Information regarding previous Infection Prevention and Control (IPC) training within the preceding 12 months was also collected as part of the participant profile.

Sample size and sampling technique

The sample size was calculated using the finite population correction formula for estimating a proportion:

$n=\frac{Z^2\times p\times q\times N}{d^2(N-1)+Z^2\times p\times q}$

where n = required sample size, N = total eligible nursing staff (234), p = anticipated prevalence of adequate knowledge/compliance (50%), q = 1 − p, d = absolute precision (15%), Z = 1.96 corresponding to a 95% confidence interval. The calculated sample size was approximately 37 participants. To compensate for potential nonresponse and improve precision, 40 nurses were ultimately included.

Participants were selected by simple random sampling (lottery method). A complete list of eligible nurses was obtained from the institutional nursing staff registry. Each eligible nurse was assigned a unique serial number, and 40 participants were selected by lottery without replacement, ensuring equal probability of selection for every eligible participant.

Eligibility criteria

Inclusion Criteria

Registered nurses involved in direct patient care, biomedical waste management, or infection prevention activities, with a minimum of one year of professional experience, who provided written informed consent to participate in the study were included.

Exclusion Criteria

Nurses who were engaged exclusively in administrative duties, were on long leave during the study period, or were unwilling to provide written informed consent were excluded from the study.

Study instrument and validation

Data were collected using a structured, self-administered, paper-based questionnaire developed using recommendations from the WHO and the CDC (Appendix A) [16,17].

The questionnaire consisted of three sections: sociodemographic and professional characteristics; knowledge of universal precautions (15 items); and compliance with universal precautions (20 items, five-point Likert scale). The questionnaire underwent content validation by three experts in Community Medicine and IPC. Pilot testing was conducted with 10 nurses not included in the final analysis to assess clarity, feasibility, and comprehensibility. Internal consistency reliability was assessed using Cronbach's alpha, which was 0.82, indicating good reliability.

Assessment of Knowledge

Knowledge regarding universal precautions was assessed using 15 multiple-choice and true/false questions covering hand hygiene, personal protective equipment, sharps safety, biomedical waste management, and transmission-based precautions.

Each correct response earned one point, while incorrect responses earned zero points, yielding a total score ranging from 0 to 15. Knowledge scores were categorized as Poor (0-5), Moderate (6-10), and Good (11-15). For negatively worded statements, the appropriate response ("False") was considered the correct answer during scoring.

Assessment of Compliance

Compliance with universal precautions was assessed using a validated 20-item five-point Likert scale with responses ranging from "Never" (1) to "Always" (5).

The total compliance score ranged from 20 to 100 and was categorized as Poor (20-46), Moderate (47-73), and Good (74-100).In addition to reporting overall compliance scores, individual compliance items were analysed separately to identify critical deficiencies in infection prevention practices, including injection safety, use of personal protective equipment, eye protection, sharps handling, and post-exposure management.

Data collection procedure

Following permission from the hospital administration, selected participants were approached personally during duty hours. The purpose of the study was explained, written informed consent was obtained, and paper-based self-administered questionnaires were distributed in person.

Participants completed the questionnaires independently without investigator assistance and returned them on the same day. Approximately 15-20 minutes were required to complete the questionnaire. Completed forms were checked immediately for completeness. Incomplete questionnaires were returned to participants for completion whenever feasible. Responses remained anonymous throughout the study.

Quality assurance and bias control

Standardized instructions were provided to all participants to ensure uniform administration. Selection bias was minimized through simple random sampling. Information bias was reduced by using a validated questionnaire, pilot testing, and anonymous self-administration, thereby minimizing interviewer and social desirability bias. Double data entry and consistency checks were performed before analysis to ensure data accuracy.

Statistical analysis

Data were entered into Microsoft Excel 2019 (Microsoft Corp., Redmond, WA, USA) and analysed using Jamovi, version 2.6.44 (The jamovi Project, Sydney, NSW, Australia). Categorical variables were summarized using frequencies and percentages. Knowledge and compliance scores were calculated according to predefined scoring criteria. Owing to the descriptive nature of the study, a relatively small sample size, and insufficient statistical power for subgroup comparisons, only descriptive statistical analyses were performed. No inferential analyses or correlation testing were undertaken.

Ethical considerations

The study protocol was reviewed and approved by the Institutional Ethics Committee of the Government Medical College, Datia (Approval No. 135/CM/GMC/IECBMHR/2026). Written informed consent was obtained from every participant before enrolment. Participation was voluntary, confidentiality and anonymity were maintained throughout the study, and no patient-related information was collected.

## Results

The study included 40 nurses working at the institution. The study population predominantly consisted of younger nurses. Although both male and female nurses were eligible for participation, all selected participants were female. Sixteen (40.0%) participants were aged 24-26 years, and 15 (37.5%) were aged 27-30 years, indicating that more than three-fourths (77.5%) belonged to the 24-30-year age group. Four (10.0%) participants were aged 31-35 years, two (5.0%) were aged 36-40 years, and three (7.5%) were aged 41-46 years. Regarding professional experience, 12 (30.0%) nurses had 1.5-2.5 years of experience, another 12 (30.0%) had 3-5 years, nine (22.5%) had 6-9 years, and seven (17.5%) had 13-22 years of professional experience (Table 1).

**Table 1:** Distribution of Study Participants according to Age and Professional Experience (n = 40) Values are presented as frequency (n) and percentage (%) of study participants according to age group and years of professional experience. Percentages were calculated based on the total study population (N = 40).

Variable	Category	Frequency (n)	Percentage (%)
Age group (years)	24–26	16	40
	27–30	15	37.5
	31–35	4	10
	36–40	2	5
	41–46	3	7.5
Years of professional experience	1.5–2.5	12	30
	3–5	12	30
	6–9	9	22.5
	13–22	7	17.5

Most participants demonstrated satisfactory knowledge regarding universal precautions. Good knowledge scores (80%-90%) were observed among 31 nurses (77.5%), while five (12.5%) achieved very good knowledge scores (>90%). Only four (10.0%) participants had average knowledge scores (<80%) (Table 2).

**Table 2:** Distribution of Knowledge Scores Regarding Universal Precautions (n = 40) Scores reflect performance in knowledge assessment; higher percentages indicate better knowledge.

Score Category	Frequency (n)	Percentage (%)
<80% (Average)	4	10
80–90% (Good)	31	77.5
>90% (Very good)	5	12.5
Total	40	100

Awareness regarding several core components of universal precautions was high among participants. All participants (100.0%) correctly identified the importance of hand hygiene between patients, the use of gloves when handling secretions, the need to avoid sharing PPE, and the requirement for additional droplet precautions when indicated. Correct responses regarding immediate hand hygiene after contamination, glove use during blood collection, changing gloves between patients, and avoiding needle recapping were reported by 39 (97.5%) participants each. Furthermore, 34 (85.0%) participants correctly recognized that the primary purpose of universal precautions is not limited solely to protecting HCWs, while 35 (87.5%) correctly identified that universal precautions should be applied to all patients rather than only those known to be infected. Knowledge regarding the use of caps during procedures with splatter risk was correctly identified by 37 (92.5%) nurses (Table 3).

**Table 3:** Distribution of Nurses According to Their Knowledge of Key Components of Universal Precautions and Infection Prevention Practices (n = 40) Values are presented as frequency (n) and percentage (%) of participants providing correct (True) or incorrect (False) responses for each knowledge variable related to universal precautions (UPs) and infection prevention practices. Percentages were calculated out of the total study population (N = 40). Frequencies represent participants providing the correct response according to the answer key. For positively worded statements, the correct response was 'True'; for negatively worded statements (e.g., 'UPs only for infected patients', 'UPs aim to protect healthcare workers', and 'No need to wash hands after glove removal'), the correct response was 'False'.

Knowledge Variables	True (n (%))	False (n (%))
Knowledge of universal precautions (UPs) measures	40 (100.0%)	0 (0.0%)
UPs only for infected patients	35 (87.5%)	5 (12.5%)
UPs aim to protect healthcare workers	6 (15.0%)	34 (85.0%)
Immediate handwashing after contamination	39 (97.5%)	1 (2.5%)
Hand hygiene between patients	40 (100.0%)	0 (0.0%)
No need to wash hands after glove removal	38 (95.0%)	2 (5.0%)
Personal protective equipment (PPE) should not be shared	40 (100.0%)	0 (0.0%)
Gloves are required for blood collection	39 (97.5%)	1 (2.5%)
Gloves are required for secretion contact	40 (100.0%)	0 (0.0%)
Gloves change between patients	39 (97.5%)	1 (2.5%)
PPE use during exposure risk	38 (95.0%)	2 (5.0%)
Use of caps in splatter risk	37 (92.5%)	3 (7.5%)
No needle recapping	39 (97.5%)	1 (2.5%)
UPs + droplet precautions when required	40 (100.0%)	0 (0.0%)
UPs + contact precautions when required	38 (95.0%)	2 (5.0%)

According to the predefined composite compliance scoring criteria, all 40 participants achieved scores within the "good compliance" category (Table 4). However, item-wise analysis revealed important variations in adherence across specific infection prevention practices. Compliance with hand hygiene was consistently high, with 36 (90.0%) nurses always performing hand hygiene between patients and 38 (95.0%) always performing hand hygiene after contamination. Similarly, high adherence was observed for glove use during wound dressing and blood cleaning (97.5%), blood collection and venipuncture (92.5%), proper sharps disposal (97.5%), and mask use (87.5%).

**Table 4:** Distribution of Nurses According to Adherence to Universal Precautions and Infection Prevention Practices in Routine Clinical Care (n = 40) Values are presented as frequency (n) and percentage (%) of participants according to the frequency of adherence to each universal precaution practice. Response categories included “Always,” “Often,” “Sometimes,” “Seldom,” and “Never.” Percentages were calculated out of the total study population (N = 40). The table reflects adherence to essential infection prevention and control practices, including hand hygiene, use of personal protective equipment, sharps disposal, injection safety, and post-exposure management. Higher frequencies in the “Always” category indicate better compliance with recommended universal precaution practices. Overall compliance categories were derived from composite compliance scores. Item-level frequencies are presented separately to identify specific deficiencies in individual universal precaution practices

Compliance Variable	Always n (%)	Often n (%)	Sometimes n (%)	Seldom n (%)	Never n (%)
Hand hygiene between patients	36 (90.0%)	4 (10.0%)	0 (0.0%)	0 (0.0%)	0 (0.0%)
Hand hygiene after glove removal	32 (80.0%)	6 (15.0%)	1 (2.5%)	0 (0.0%)	1 (2.5%)
Hand hygiene after contamination	38 (95.0%)	2 (5.0%)	0 (0.0%)	0 (0.0%)	0 (0.0%)
Gloves for blood collection	37 (92.5%)	2 (5.0%)	1 (2.5%)	0 (0.0%)	0 (0.0%)
Gloves for urine/feces contact	35 (87.5%)	4 (10.0%)	0 (0.0%)	0 (0.0%)	1 (2.5%)
Gloves for non-intact skin	33 (82.5%)	5 (12.5%)	1 (2.5%)	0 (0.0%)	1 (2.5%)
Gloves for mucous membrane	38 (95.0%)	1 (2.5%)	1 (2.5%)	0 (0.0%)	0 (0.0%)
Gloves for airway discharge	38 (95.0%)	1 (2.5%)	1 (2.5%)	0 (0.0%)	0 (0.0%)
Gloves during injections	22 (55.0%)	10 (25.0%)	2 (5.0%)	2 (5.0%)	4 (10.0%)
Gloves during wound dressing	39 (97.5%)	1 (2.5%)	0 (0.0%)	0 (0.0%)	0 (0.0%)
Gloves during blood cleaning	39 (97.5%)	1 (2.5%)	0 (0.0%)	0 (0.0%)	0 (0.0%)
Gloves during venipuncture	37 (92.5%)	2 (5.0%)	1 (2.5%)	0 (0.0%)	0 (0.0%)
Gloves during blood sample handling	37 (92.5%)	3 (7.5%)	0 (0.0%)	0 (0.0%)	0 (0.0%)
Mask use	35 (87.5%)	1 (2.5%)	3 (7.5%)	0 (0.0%)	1 (2.5%)
Eye protection use	30 (75.0%)	1 (2.5%)	0 (0.0%)	5 (12.5%)	4 (10.0%)
Apron use	26 (65.0%)	4 (10.0%)	4 (10.0%)	5 (12.5%)	1 (2.5%)
Caps/shoe covers use	27 (67.5%)	0 (0.0%)	7 (17.5%)	5 (12.5%)	1 (2.5%)
Avoid recapping needles	31 (77.5%)	1 (2.5%)	3 (7.5%)	1 (2.5%)	4 (10.0%)
Proper sharps disposal	39 (97.5%)	1 (2.5%)	0 (0.0%)	0 (0.0%)	0 (0.0%)
Post-exposure management	33 (82.5%)	2 (5.0%)	1 (2.5%)	0 (0.0%)	4 (10.0%)

Despite these favourable overall compliance scores, several critical deficiencies were identified. Only 22 (55.0%) nurses consistently used gloves during injections, while regular use of aprons and caps/shoe covers was reported by only 26 (65.0%) and 27 (67.5%) participants, respectively. Eye protection showed comparatively poor adherence, with only 30 (75.0%) nurses reporting consistent use, whereas nine (22.5%) reported seldom or never using eye protection during procedures associated with splash risk. Furthermore, four (10.0%) nurses reported never avoiding needle recapping, and four (10.0%) did not consistently follow recommended post-exposure management following sharps injuries. These findings indicate that although overall compliance scores were favourable, important deficiencies persisted in specific high-risk infection prevention practices.

## Discussion

Universal precautions are an essential part of preventive medicine, helping to minimize healthcare-associated infections and occupational exposure among HCWs. In a study conducted at a tertiary care teaching hospital in Datia, nurses demonstrated good knowledge of universal precautions, with 77.5% having adequate knowledge of basic principles, including hand hygiene, glove use, and proper sharps disposal. Adegboye et al. (2018) reported that although 86% of HCWs had good knowledge of infection control, only 32.5% correctly practiced the six-step handwashing technique, and fewer than 14% had received formal infection control training, indicating a marked knowledge-practice gap [18]. Onyedibe et al. (2020) found an overall hand hygiene compliance of only 31% among HCWs in a tertiary hospital in northern Nigeria, with poor compliance largely attributed to inadequate hand hygiene facilities, limited training, and insufficient institutional support [19].

Despite adequate knowledge, critical gaps were found in the understanding and implementation of universal precautions. Most nurses (85.0%) correctly recognized that the primary purpose of universal precautions is not limited solely to protecting HCWs, although 12.5% incorrectly believed that universal precautions should be applied only to infected patients. Sagar et al. (2020) reported that although healthcare providers demonstrated good hand hygiene knowledge (73.8% among consultants and 71.6% among residents), compliance remained considerably lower (49.5% and 39.1%, respectively), highlighting a significant knowledge-practice gap mainly due to lack of awareness, time constraints, and heavy workload [20]. Arora et al. (2021) demonstrated that a structured training intervention significantly improved healthcare providers' knowledge, attitudes, preparedness, and practices, supporting the effectiveness of regular training programmes in strengthening healthcare practices [21]. In the present study, compliance was high for hand hygiene (90%-95%) and glove use during high-risk procedures (92.5-97.5%), findings consistent with observations by Alhumaid et al. (2021), who reported higher adherence among nurses than among other HCWs [10].

The majority of participants in the present study were young nurses with relatively limited professional experience. Although overall knowledge of universal precautions was satisfactory, important deficiencies were observed in specific infection-prevention practices, particularly injection safety, use of PPE, eye protection, and post-exposure management. These findings suggest that adequate knowledge alone may not translate into consistent adherence to all components of universal precautions, highlighting the need for regular training, supportive supervision, and continuous monitoring of infection prevention practices.

However, compliance was suboptimal for injection safety (55%), use of protective aprons, caps, and shoe covers (65%-67.5%), and post-exposure practices following sharps injuries (10% non-adherence). Ezike et al. (2022) reported that infection prevention practices among nurses were suboptimal, with poor decontamination of high-touch surfaces and inadequate availability of PPE in only 50% of medical wards, emphasizing the need for continuous training and reinforcement of infection prevention and control measures [14]. Brooks et al. (2021), in a systematic review of 56 studies, identified inadequate PPE availability, discomfort, poor communication of infection control guidance, lack of managerial support, and insufficient training as major barriers to HCWs' compliance with infection control measures, while supervision and role-specific education were associated with improved adherence [22]. King et al. (2023) reported that 71% of home care providers consistently adhered to facial protective equipment recommendations, with higher adherence to mask use (89%) than eye protection (73%), and found that perceived PPE efficacy, knowledge of recommended use, perceived occupational risk, and fewer personal barriers were significant predictors of adherence [23].

The identified deficiencies in universal precautions represent missed opportunities to prevent healthcare-associated infections and protect HCWs from occupational exposure. Tan et al. (2020), in a systematic review of studies from low- and middle-income countries, reported that implementation of tuberculosis infection prevention and control measures was frequently overestimated by self-report, while inadequate staff training, poor managerial support, limited availability of respirators, and low risk perception were major barriers contributing to a persistent knowledge-action gap [24]. Similarly, Vasan et al. (2017), in a comparative review of 40 studies from low- and middle-income countries, identified supportive supervision, mentoring, quality improvement methods, coaching, and performance tools as key strategies for improving HCW performance, emphasizing the importance of structured supervision and continuous performance improvement [25]. Consistent with these findings, our study highlights the need for regular competency-based training, supportive supervision, and periodic compliance audits to strengthen adherence to universal precautions. Improved compliance with these measures is essential for safe healthcare delivery and aligns with the emphasis by Lahariya (2020) that effective infection prevention and control is a key policy priority for strengthening health and wellness centers and the broader primary healthcare system in India [26].

The findings support strengthening institutional infection prevention programmes through regular competency-based training, adequate PPE availability, and periodic compliance audits.

Limitations

The study was limited by a small sample size (n = 40) and its conduct at a single institution, which may affect generalizability. The cross-sectional design does not allow causal inference. Data were self-reported, introducing potential recall and social desirability bias, and actual practices were not directly observed. Additionally, the use of a self-designed questionnaire and the limited assessment of confounding factors may affect the comparability and depth of the analysis. The composite compliance scoring system may have overestimated overall compliance because high scores in routine practices masked deficiencies in individual high-risk practices; therefore, item-level findings should be interpreted alongside overall compliance scores.

## Conclusions

Although nurses demonstrated good overall knowledge of universal precautions, important deficiencies persisted in specific practices, particularly glove use during injections, eye protection, use of aprons and caps/shoe covers, safe needle handling, and post-exposure management. Regular competency-based training, continuous monitoring, and improved availability of PPE are recommended to bridge the knowledge-practice gap and strengthen infection prevention practices.

## Appendices

Appendix A 

**Table 5:** Validated Structured Questionnaire for Assessment of Sociodemographic Characteristics, Knowledge, and Compliance Regarding Infection Prevention and Control Practices Among Nurses Structured validated questionnaire used for assessing sociodemographic characteristics, knowledge, and compliance regarding infection prevention and control practices among nurses at Government Medical College, Datia. Section A includes participants’ sociodemographic and professional details. Section B assesses knowledge regarding infection prevention and control practices using 15 multiple-choice questions. Each correct response was awarded 1 mark and incorrect responses 0 marks, with total scores categorized as Poor (0–5), Moderate (6–10), and Good (11–15) knowledge. Section C evaluates compliance with infection prevention and control practices using a 20-item five-point Likert scale ranging from “Never” (1) to “Always” (5). Total compliance scores ranged from 20–100 and were classified as Poor (20–46), Moderate (47–73), and Good (74–100) compliance. The questionnaire was developed based on WHO and CDC infection prevention guidelines [16,17] and validated through expert review and pilot testing.

Section A: Sociodemographic and Professional Profile						
Q. No.	Question	Response Options				
1	Age (in completed years)	__________________				
2	Gender	☐ Male ☐ Female ☐ Other				
3	Educational qualification	☐ GNM ☐ B.Sc. Nursing ☐ Post Basic B.Sc. ☐ M.Sc. Nursing				
4	Current designation	☐ Staff Nurse ☐ Nursing Officer ☐ Sister In-charge ☐ Other __________				
5	Department/Unit	☐ ICU ☐ OT ☐ Medicine ☐ Surgery ☐ Pediatrics ☐ Obstetrics & Gynecology ☐ Emergency ☐ Other __________				
6	Total years of professional experience	☐ <1 year ☐ 1–5 years ☐ 6–10 years ☐ >10 years				
7	Have you received infection control training during the last 12 months?	☐ Yes ☐ No				
8	Have you attended any hand hygiene workshop/CME previously?	☐ Yes ☐ No				
9	Average duty hours/day	☐ <6 hours ☐ 6–8 hours ☐ >8 hours				
10	Have you ever experienced a needle-stick injury?	☐ Yes ☐ No				
Section B: Knowledge Regarding Infection Prevention and Control						
Instructions						
Tick (☑) the most appropriate answer.						
Q. No.	Question	Response Options				
1	What is the single most effective method to prevent hospital-acquired infections?	☐ Vaccination ☐ Hand hygiene ☐ Antibiotics ☐ Isolation				
2	Alcohol-based hand rub should preferably be used when hands are:	☐ Visibly dirty ☐ Not visibly soiled ☐ Contaminated with blood ☐ After toilet use				
3	The recommended minimum duration for handwashing with soap and water is:	☐ 5 seconds ☐ 10 seconds ☐ 20 seconds ☐ 1 minute				
4	Standard precautions should be applied to:	☐ All patients ☐ ICU patients only ☐ Infectious patients only ☐ Surgical patients only				
5	Which of the following is included under personal protective equipment (PPE)?	☐ Gloves ☐ Masks ☐ Gowns ☐ All of the above				
6	Used sharps should be discarded in:	☐ Black container ☐ Red bag ☐ White puncture-proof container ☐ General waste bin				
7	Biomedical waste segregation should occur at:	☐ Central storage room ☐ Nursing station ☐ Point of generation ☐ Laboratory only				
8	Which infection may spread through needle-stick injury?	☐ Hepatitis B ☐ Malaria ☐ Dengue ☐ Typhoid				
9	Gloves should be changed:	☐ Once per shift ☐ Between patients ☐ Daily ☐ Only when damaged				
10	Recapping used needles is:	☐ Recommended ☐ Discouraged ☐ Mandatory ☐ Safe practice				
11	N95 masks are mainly recommended for prevention of:	☐ Droplet infection ☐ Airborne infection ☐ Waterborne infection ☐ Contact infection				
12	Hand hygiene should be performed before and after patient contact.	☐ True ☐ False				
13	Blood and body fluid spills should be cleaned using:	☐ Plain water ☐ Detergent only ☐ Appropriate disinfectant ☐ Dry cloth				
14	Isolation precautions are required for patients with highly communicable diseases.	☐ True ☐ False				
15	Hospital-acquired infections are also known as:	☐ Vector-borne infections ☐ Nosocomial infections ☐ Endemic infections ☐ Community infections				
Section C: Compliance with Infection Prevention and Control Practices						
Instructions						
Please tick (☑) the option that best describes your routine practice.						
Response	Score					
Never	1					
Rarely	2					
Sometimes	3					
Often	4					
Always	5					
Q. No.	Statement	Never	Rarely	Sometimes	Often	Always
1	I wash my hands before touching a patient.	☐	☐	☐	☐	☐
2	I wash my hands after touching a patient.	☐	☐	☐	☐	☐
3	I use alcohol-based hand rub when indicated.	☐	☐	☐	☐	☐
4	I wear gloves during contact with blood/body fluids.	☐	☐	☐	☐	☐
5	I change gloves between patient contacts.	☐	☐	☐	☐	☐
6	I wear masks during procedures with infection risk.	☐	☐	☐	☐	☐
7	I use gowns/aprons during high-risk procedures.	☐	☐	☐	☐	☐
8	I follow biomedical waste segregation guidelines.	☐	☐	☐	☐	☐
9	I discard sharps immediately after use.	☐	☐	☐	☐	☐
10	I avoid recapping used needles.	☐	☐	☐	☐	☐
11	I follow standard precautions for every patient.	☐	☐	☐	☐	☐
12	I disinfect reusable medical equipment after use.	☐	☐	☐	☐	☐
13	I clean blood/body fluid spills promptly using disinfectants.	☐	☐	☐	☐	☐
14	I report needle-stick injuries immediately.	☐	☐	☐	☐	☐
15	I participate in infection control training programs.	☐	☐	☐	☐	☐
16	I follow isolation precautions when indicated.	☐	☐	☐	☐	☐
17	I dispose contaminated materials appropriately.	☐	☐	☐	☐	☐
18	I maintain aseptic precautions during procedures.	☐	☐	☐	☐	☐
19	I encourage colleagues to comply with IPC practices.	☐	☐	☐	☐	☐
20	I consistently follow hospital infection control protocols.	☐	☐	☐	☐	☐

## References

[1] Kuhar DT, Carrico RM, Cox K (2026). Infection control in healthcare personnel: infrastructure and routine practices. Atlanta (GA): Centers for Disease Control and Prevention.

[2] Centers for Disease Control and Prevention (CDC) (1987). Recommendations for prevention of HIV transmission in health-care settings. MMWR Morb Mortal Wkly Rep.

[3] (2026). HIV/AIDS surveillance report: U.S. AIDS cases reported through June 1993. https://stacks.cdc.gov/view/cdc/32522.

[4] Luo Y, He GP, Zhou JW, Luo Y (2010). Factors impacting compliance with standard precautions in nursing, China. Int J Infect Dis.

[5] Raoofi S, Pashazadeh Kan F, Rafiei S (2023). Global prevalence of nosocomial infection: a systematic review and meta-analysis. PLoS One.

[6] Mathur P (2023). Need for national-level surveillance of HAIs in India. J Clin Infect Dis Soc.

[7] Murhekar MV, Kumar CG (2022). Health-care-associated infection surveillance in India. Lancet Glob Health.

[8] Adesina KA, Aselebe M, Afolalu O, Oyekale R (2022). Awareness and practices of standard safety precaution amongst nurses toward prevention of nosocomial infections in selected hospital in Osun State. Int J Innov Res Adv Stud.

[9] Agarwal A, Ranjan P, Saraswat A (2021). Are health care workers following preventive practices in the COVID-19 pandemic properly? - A cross-sectional survey from India. Diabetes Metab Syndr.

[10] Alhumaid S, Al Mutair A, Al Alawi Z (2021). Knowledge of infection prevention and control among healthcare workers and factors influencing compliance: a systematic review. Antimicrob Resist Infect Control.

[11] Constantinou D, Leontiou I, Mpouzika M, Michail K, Middletton N, Merkouris A (2024). Correction: health care workers' knowledge and perceptions on WHO hand hygiene guidelines, and the perceived barriers to compliance with hand hygiene in Cyprus. BMC Nurs.

[12] Deressa W, Worku A, Abebe W, Gizaw M, Amogne W (2021). Risk perceptions and preventive practices of COVID-19 among healthcare professionals in public hospitals in Addis Ababa, Ethiopia. PLoS One.

[13] Obirikorang C, Opoku SK, Obirikorang Y (2019). Awareness and occupational exposures to needlestick injuries among healthcare workers: a quantitative assessment in a Ghanaian metropolis. Glob J Qual Saf Healthc.

[14] Ezike OC, Odikpo LC, Onyia EN (2022). Risk perception, risk involvement/exposure and compliance to preventive measures to COVID-19 among nurses in a tertiary hospital in Asaba, Nigeria. Int J Afr Nurs Sci.

[15] Coumare VN, Pawar SJ, Manoharan PS (2021). COVID-19 pandemic-frontline experiences and lessons learned from a tertiary care teaching hospital at a suburban location of southeastern India. Front Public Health.

[16] (2016). Guidelines on core components of infection prevention and control programmes at the national and acute health care facility level. https://www.who.int/publications/i/item/9789241549929.

[17] (2024). CDC’s core infection prevention and control practices for safe healthcare delivery in all settings. https://www.cdc.gov/infection-control/hcp/core-practices/index.html.

[18] Adegboye MB, Zakari S, Ahmed BA, Olufemi GH (2018). Knowledge, awareness and practice of infection control by health care workers in the intensive care units of a tertiary hospital in Nigeria. Afr Health Sci.

[19] Onyedibe KI, Shehu NY, Pires D (2020). Assessment of hand hygiene facilities and staff compliance in a large tertiary health care facility in northern Nigeria: a cross sectional study. Antimicrob Resist Infect Control.

[20] Sagar M, Sharma S, Chaudhary A, Sharma S (2020). A mixed-method study to assess the knowledge-practice gap regarding hand hygiene among healthcare providers in a tertiary care hospital. J Anaesthesiol Clin Pharmacol.

[21] Arora S, Rege S, Bhate-Deosthali P, Thwin SS, Amin A, García-Moreno C, Meyer SR (2021). Knowledge, attitudes and practices of health care providers trained in responding to violence against women: a pre- and post-intervention study. BMC Public Health.

[22] Brooks SK, Greenberg N, Wessely S, Rubin GJ (2021). Factors affecting healthcare workers' compliance with social and behavioural infection control measures during emerging infectious disease outbreaks: rapid evidence review. BMJ Open.

[23] King EC, Zagrodney KA, McKay SM, Holness DL, Nichol KA (2023). Determinants of nurse's and personal support worker's adherence to facial protective equipment in a community setting during the COVID-19 pandemic in Ontario, Canada: a pilot study. Am J Infect Control.

[24] Tan C, Kallon II, Colvin CJ, Grant AD (2020). Barriers and facilitators of tuberculosis infection prevention and control in low- and middle-income countries from the perspective of healthcare workers: a systematic review. PLoS One.

[25] Vasan A, Mabey DC, Chaudhri S, Brown Epstein HA, Lawn SD (2017). Support and performance improvement for primary health care workers in low- and middle-income countries: a scoping review of intervention design and methods. Health Policy Plan.

[26] Lahariya C (2020). Health & wellness centers to strengthen primary health care in India: concept, progress and ways forward. Indian J Pediatr.

